# Generation of Active Protein Phosphatase 2A Is Coupled to Holoenzyme Assembly 

**DOI:** 10.1371/journal.pbio.0050155

**Published:** 2007-06-05

**Authors:** Hans Hombauer, David Weismann, Ingrid Mudrak, Claudia Stanzel, Thomas Fellner, Daniel H Lackner, Egon Ogris

**Affiliations:** Department of Medical Biochemistry, Max F. Perutz Laboratories, Medical University of Vienna, Vienna, Austria; University of California San Francisco, United States of America

## Abstract

Protein phosphatase 2A (PP2A) is a prime example of the multisubunit architecture of protein serine/threonine phosphatases. Until substrate-specific PP2A holoenzymes assemble, a constitutively active, but nonspecific, catalytic C subunit would constitute a risk to the cell. While it has been assumed that the severe proliferation impairment of yeast lacking the structural PP2A subunit, TPD3, is due to the unrestricted activity of the C subunit, we recently obtained evidence for the existence of the C subunit in a low-activity conformation that requires the RRD/PTPA proteins for the switch into the active conformation. To study whether and how maturation of the C subunit is coupled with holoenzyme assembly, we analyzed PP2A biogenesis in yeast. Here we show that the generation of the catalytically active C subunit depends on the physical and functional interaction between RRD2 and the structural subunit, TPD3. The phenotype of the *tpd3*Δ strain is therefore caused by impaired, rather than increased, PP2A activity. TPD3/RRD2-dependent C subunit maturation is under the surveillance of the PP2A methylesterase, PPE1, which upon malfunction of PP2A biogenesis, prevents premature generation of the active C subunit and holoenzyme assembly by counteracting the untimely methylation of the C subunit. We propose a novel model of PP2A biogenesis in which a tightly controlled activation cascade protects cells from untargeted activity of the free catalytic PP2A subunit.

## Author Summary

Multisubunit enzymes, such as protein phosphatase 2A, consist of a catalytic subunit and one of several regulatory subunits that are responsible for substrate specificity. Whereas this molecular architecture enables the assembly of a few components into many different substrate-specific enzymes, it possesses an inherent danger in the form of the uncomplexed catalytic subunit with its unspecific phosphatase activity. Until substrate-specific complexes assemble, the catalytic subunit would constitute a risk to the cell if no control mechanisms existed. We recently obtained evidence for the existence of the catalytic subunit in a low-activity conformation that requires an activator for the switch into the active conformation. This requirement suggested that the existing model of protein phosphatase 2A biogenesis was incomplete, because it could not explain how the activity of the catalytic subunit is kept in check until it is assembled with the substrate-targeting subunits. In this study, we provide evidence that the generation of the active catalytic subunit is coupled with and regulated by holoenzyme assembly. We propose a novel model of protein phosphatase biogenesis in which a tightly controlled activation cascade protects cells from the potential risk of unspecific dephosphorylation events.

## Abstract 3

Analysis of protein phosphatase 2A (PP2A) biogenesis in yeast suggests that a tightly controlled activation cascade, involving an interaction between the protein RRD2 and the structural subunit TPD3, protects cells from untargeted activity of the free catalytic PP2A subunit.

## Introduction

An estimated one third of the cellular proteome is reversibly phosphorylated on specific serine/threonine residues, indicating an involvement of phosphorylation in many, if not all, cellular processes. The rapid reversibility and the selective functional effects of phosphorylation on substrate functions contribute to its critical importance in signaling cascades. The net phosphorylation of any given regulatory site is determined both by the enzymes catalyzing the phosphorylation reaction—the protein serine/threonine kinases (PSTKs)—and by the enzymes catalyzing the dephosphorylation reaction—the protein serine/threonine phosphatases (PSTPs). The molecular architecture that allows these two opposing enzyme classes to achieve substrate specificity has evolved very differently in each class. This is reflected in the number of genes coding for PSTKs (~430) versus only about 30 genes encoding catalytic subunits of the PSTPs [1]. Most PSTKs are multidomain, single-chain enzymes containing a catalytic and a substrate regulatory domain. PSTPs, however, are multisubunit enzymes in which the catalytic and regulatory subunits are encoded by different genes. Thus the assembly of different combinations of subunits to produce phosphatase holoenzymes with different substrate specificities compensates for the low number of PSTP catalytic subunits.

A prime example of the PSTP multisubunit architecture is protein phosphatase 2A (PP2A). PP2A in vivo comprises a large number of heterodimeric and -trimeric complexes, all of which contain a catalytic C subunit. In the majority of PP2A holoenzymes, the C subunit is bound to the regulatory A subunit, which serves as a scaffold for the interaction with one of several B-type subunits. The B-type subunits are responsible for the substrate specificity and intracellular localization of the PP2A holoenzymes. Four B-type subunit families have been identified in mammalian cells, each family consisting of several highly related isoforms. In theory, more than 72 heterotrimeric holoenzymes can be assembled from the large pool of PP2A subunit isoforms [2].

A major determinant of holoenzyme composition in vivo is the availability of specific regulatory subunits, which in turn depends on their spatially and temporally regulated expression. Whereas some holoenzymes only form in certain developmental stages, others are present at all times and constitute “housekeeping” PP2A complexes. Another important determinant seems to be the stoichiometric balance of C, A, and B-type subunits. Down-regulation of the PP2A C or A subunit by RNA interference (RNAi) in vivo leads to the rapid degradation of the non-targeted subunits, suggesting that PP2A subunits are stabilized in the holoenzyme, but are instable as monomeric subunits [3,4]. Consequently, native PP2A isolated from various tissues consists of dimeric and trimeric complexes, but does not contain the free monomeric C subunit [5]. How the absolute levels of PP2A subunits and, therefore, of holoenzymes are adjusted to the cell's respective requirements is unknown, but the catalytic PP2A subunit with its strictly controlled expression levels seems to play a central role in this regulatory circuit.

Monomeric C subunits isolated from in vivo–assembled PP2A holoenzymes possess promiscuous and high activity in vitro [5,6]. Complex formation with the regulatory A and B-type subunits suppresses nonspecific C subunit activity, but increases the substrate affinity, as reflected by the decreased maximum velocity (Vmax) and Km [7]. Tight control of C subunit levels provides one explanation for how cells prevent the consequences of having free and unregulated C subunit [8]. However, it leaves open the question of how the catalytic activity of the newly translated C subunit (or transiently free C subunit, if recycling occurs) is kept in check until holoenzyme assembly can occur. Holoenzyme assembly is promoted by reversible methylation of the C subunit carboxyterminal leucine, which increases its affinity for the A and B-type subunits [9,10]. Deletion of *protein phosphatase methyltransferase 1 (PPM1),* encoding the yeast protein phosphatase methyltransferase solely responsible for PP2A methylation, not only negatively affects the interaction between the yeast C subunit homologs, PPH21 and PPH22, and the scaffold subunit, TPD3, but also affects the interaction with the B subunits, CDC55 and RTS1 [9,10]. The recently solved structures of a heterotrimeric PP2A complex suggest charge neutralization of the C subunit C-terminal carboxyl group as a potential cause for the affinity increase by methylation [11,12]. Although carboxymethylation is required for stable complex formation of PP2A holoenzymes in vivo [9,10], this does not seem to be the case in vitro [12], indicating a more complex role for C subunit methylation in vivo. Interestingly, deletion of *PPM1* also reduces the catalytic activity of C subunit [13]. The discrepancy between the high catalytic activity of the free C subunit isolated in vitro from pre-existing holoenzymes and the decreased catalytic activity of the C subunit from a *ppm1*Δ strain with a hampered holoenzyme formation suggests that holoenzyme assembly might be coupled to the generation of the catalytically active C subunit and that methylation might play an important role in both processes.

Recently, we obtained experimental evidence for a conserved mechanism to generate active and specific C subunit in vivo [13]. Deletion of *rapamycin-resistant deletion 1* and *2 (RRD1* and *RRD2),* encoding the yeast homologs of the PP2A phosphatase activator (PTPA), produces a catalytic subunit with a conformationally relaxed active site, as indicated by its low catalytic activity towards phospho-serine/phospho-threonine (P-Ser/P-Thr) substrates, an altered substrate specificity, and its metal dependence. RRD2 stably interacts with the PP2A C subunit and, when expressed ectopically in the *rrd1*Δ*/rrd2*Δ strain, partially rescues the catalytic activity of the PP2A C subunit. RRD1, on the other hand, seems to be required for the generation of active SIT4, a PP2A-related phosphatase. Our data suggested a mechanism by which cells may avoid the risk of free and unregulated catalytic phosphatase subunits, namely the synthesis of a low-activity form that requires a functional interaction with an activator protein for full activity. However, this leaves open the question how the biogenesis of the catalytic subunit is coupled to the methylation-dependent PP2A holoenzyme assembly.

Here we present evidence that the scaffolding PP2A subunit, TPD3, interacts functionally and physically with RRD/PTPA in the generation of active and specific PP2A in vivo. Further, the PP2A methylesterase, PPE1, plays a multifaceted role, since deletion of *PPE1* in an *rrd1*Δ*/rrd2*Δ strain increases holoenzyme assembly with the B-type subunit CDC55 without generation of the active C subunit, whereas deletion of *PPE1* in a *tpd3*Δ strain restores C subunit activity despite a failure of holoenzyme formation. Considered in detail, our results support a model in which C subunit biogenesis and PP2A holoenzyme assembly depend on a series of interlocking steps that proceed under surveillance of the PP2A methylesterase PPE1 by keeping in check the untimely methylation of the C subunit.

## Results

### Loss of TPD3 Leads to a Decreased Serine/Threonine Phosphatase Activity and Altered Substrate Specificity of the PP2A C Subunit

The scaffolding A subunit is a prerequisite for the assembly of most PP2A holoenzymes. In the absence of the A subunit, TPD3, yeast proliferate poorly and have defects in tRNA transcription at high temperatures and in cytokinesis at low temperatures [14]. This latter phenotype is similar to the one caused by overexpression of the PP2A catalytic subunit [15]. It has therefore been assumed, that the unrestricted and untargeted catalytic activity of the C subunit is responsible for the observed phenotype in the absence of TPD3. Our recent work indicated, however, that the PP2A C subunit can exist in a low-activity form that requires the functional interaction with RRD/PTPA for the switch into an active P-Ser/P-Thr–specific enzyme [13]. Thus we asked whether the observed phenotype of the *tpd3*Δ strain, a strain impaired in holoenzyme assembly, is indeed caused by the unrestricted activity of the free C subunit.

**Figure 1:**
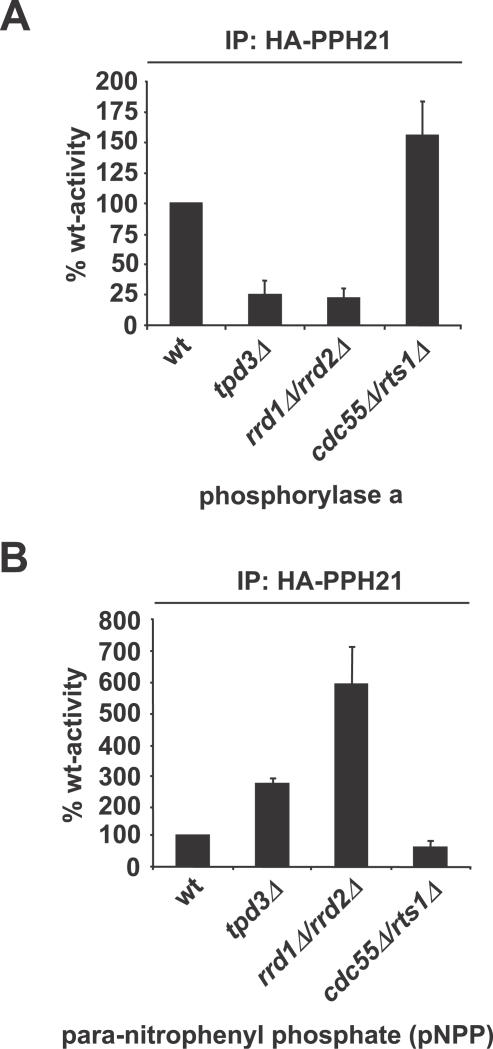
Loss of TPD3 Leads to a Decreased Serine/Threonine Phosphatase Activity and Altered Substrate Specificity of the PP2A C Subunit The catalytic activity of anti-HA immunoprecipitates from lysates of wild-type (wt), *tpd3*Δ, *rrd1*Δ*/rrd2*Δ, and *cdc55*Δ*/rts1*Δ cells expressing HA-tagged PPH21 was analyzed by phosphatase assays towards (A) phosphorylase a (*n* = 12, mean ± standard deviation) or (B) para-nitrophenyl phosphate, pNPP (*n* = 4, mean ± standard deviation). An additional background measurement (corresponding to the wild-type strain containing the empty vector pYX142 was included for every assay (A and B) and subtracted from each measuring point (see Materials and Methods for more details).

To analyze PP2A activity under these diverse conditions, we expressed hemagglutinin (HA)-tagged PPH21 or HA-tagged PPH22 in the wild-type, *tpd3*Δ, and *rrd1*Δ*/rrd2*Δ strains, and in a *cdc55*Δ*/rts1*Δ strain, which lacks the known B-type subunits of yeast. Anti–HA-tag immunoprecipitates were tested for catalytic activity towards the P-Ser/P-Thr substrate phosphorylase a (Figures 1A and S1A). Contrary to the prediction, the catalytic activity of PPH21 or PPH22 from a strain lacking the TPD3 subunit was decreased to approximately 25% or approximately 18%, respectively, of the wild-type activity. The extent of the decrease was similar for substrate concentrations ranging from 1 μM phosphorylase a to 100 μM, a concentration 20-fold above the reported Km value for phosphorylase a (at which concentration the velocity converges to the Vmax of the enzyme) (Figure S2) [16]. A decreased catalytic activity, however, was not a general feature of strains lacking PP2A regulatory subunits. Deletion of the two known B-type subunits in yeast, CDC55 and RTS1, did not impair C subunit activity towards phosphorylase a. In addition, we analyzed the catalytic activity of endogenous C subunit immunoprecipitates from lysates of a wild-type or a *tpd3*Δ strain that did not express ectopically HA-tagged PPH21 or PPH22 (Figure S3B). The endogenous C subunit activity was also severely impaired towards phosphorylase a in the *tpd3*Δ strain. Thus, TPD3 seems to fulfill functions in addition to serving as the scaffold for the interaction with B-type subunits.

The C subunit in the *rrd1*Δ*/rrd2*Δ strain shows a decreased P-Ser/P-Thr–specific activity. Strikingly, however, it gains high activity towards para-nitrophenyl phosphate (pNPP), a small-molecule substrate with a bulky phospho-tyrosine–like group [13] (Figure 1B). Both C subunits, PPH21 as well as PPH22, displayed several-fold higher activities towards pNPP in the *tpd3*Δ strain than in the wild-type strain (Figures 1B and S1B). Thus, loss of TPD3 led to the generation of C subunits with altered catalytic properties, indicating a role for TPD3 in C subunit biogenesis.

### Decreased Catalytic Activity Is Intrinsic to the C Subunit

**Figure 2:**
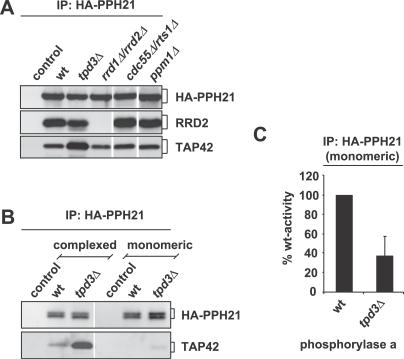
Decreased Catalytic Activity Is Intrinsic to the C Subunit (A) Anti–HA-tag immunoprecipitates from lysates of wild-type (wt) cells containing an empty vector (control) or wild-type, *tpd3*Δ, *rrd1*Δ*/rrd2*Δ, *cdc55*Δ*/rts1*Δ, and *ppm1*Δ cells expressing HA-tagged PPH21 were analyzed by SDS-PAGE and immunoblotting. The blots were sequentially incubated with specific antibodies against HA-tag, RRD2, and TAP42. Lanes 4–6 were not adjacent on the original blot. (B) Monomeric C subunits from the wild-type and the *tpd3*Δ strains were obtained by immunoprecipitation from lysates in which protein complexes had been disrupted by a basic pH shift, followed by neutralization (monomeric), or from control untreated lysates (complexed) (see Materials and Methods for details). The HA-PPH21 immunoprecipitates were analyzed by SDS-PAGE and immunoblotting. (C) An aliquot of the monomeric HA-PPH21 immunoprecipitates was tested by phosphatase assays towards phosphorylase a (*n* = 3).

The decreased C subunit activity and altered substrate specificity in the *tpd3*Δ strain were reminiscent of the hampered PP2A activity in a strain lacking RRD1 and RRD2 [13]. RRD2 is known to form stable interactions with the yeast C subunits and to be significantly more active in vivo on the PP2A catalytic subunit than RRD1 is. We asked whether the interaction of the C subunit with RRD2 was affected by deletion of *TPD3*. Anti–HA-tag immunoprecipitates from lysates of the wild-type, *tpd3*Δ, *rrd1*Δ*/rrd2*Δ, *cdc55*Δ*/rts1*Δ, and *ppm1*Δ strains were analyzed for the presence of RRD2 (Figure 2A). The interaction of HA-tagged PPH21 with RRD2 was only slightly reduced in the *tpd3*Δ strain (64% ± 26% of wild-type strain level, *n* = 10), but the levels of associated TAP42, a highly conserved and essential subunit of the PP2A family, increased several-fold, as has been shown by others [17]. Competency for TAP42 binding indicated that deletion of *TPD3* did not lead to formation of denatured C subunit. The increased levels of TAP42:C subunit complexes in a *tpd3*Δ strain have been attributed to the lack of PP2A heterotrimers, which may promote the dissociation of the TAP42:C subunit complex by dephosphorylation of TAP42 [18]. However, in contrast to the *tpd3*Δ strain, the level of TAP42:C subunit complexes remained unchanged in the *cdc55*Δ*/rts1*Δ strain, indicating that the presence or absence of heterotrimeric PP2A does not alter the stability of the TAP42:C subunit complexes.

Genetic evidence indicates an inhibitory role for TAP42 on phosphatase activity [19]. To exclude the possibility that the decreased catalytic activity and altered substrate specificity of the C subunit isolated from the *tpd3*Δ strain is simply due to formation of the TAP42:C subunit complex or binding of an unknown inhibitor, we measured the catalytic activity of immunoprecipitated HA-tagged PPH21 that was stripped of associated proteins, including TAP42, at alkaline pH (Figure 2B and 2C). To verify the monomeric nature of the C subunit, we analyzed pH-treated lysates from a wild-type or *tpd3*Δ strain by sucrose gradient sedimentation/centrifugation (Figure S4). The pH treatment shifted HA-tagged PPH21 to lower molecular weight fractions, indicating dissociation of C subunit complexes and the presence of monomeric C subunits. Compared with the wild-type strain, the “free” C subunit from the *tpd3*Δ strain was still impaired in catalytic activity (Figure 2C), indicating that the defect is intrinsic to the C subunit and not caused by the increased binding of TAP42 or any other inhibitor. C subunit activity was decreased even at high substrate concentrations (Figure S5), confirming the results obtained with the complexed C subunit and indicating a defect in the catalytic mechanism. In summary, loss of TPD3 led to alterations of C subunit activity and specificity, which are most probably due to changes at the active site that are reminiscent of the alterations observed in the absence of RRD1/RRD2.

### RRD2 Interacts Physically and Functionally with TPD3

**Figure 3:**
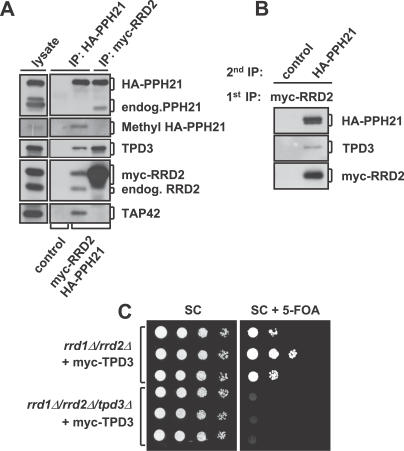
RRD2 Interacts Physically and Functionally with TPD3 (A) HA-PPH21 or myc-RRD2 immunoprecipitates (IP) from lysates of wild-type (wt) cells co-expressing HA-PPH21 and myc-RRD2 were analyzed by SDS-PAGE and immunoblotting. The blots were sequentially incubated with anti-methyl PP2A/PPH21 (2A10), rabbit polyclonal anti-TPD3, anti-RRD2, anti-TAP42, and anti-PPH21 antibody. endog., endogenous. (B) Anti–Myc-tag immunoprecipitates from lysates of wild-type cells co-expressing myc-RRD2 and HA-PPH21 were eluted by peptide competition and re-immunoprecipitated via HA-PPH21 (see Materials and Methods for details). “Control” indicates a wild-type strain containing both empty vectors. The re-immunoprecipitates were analyzed by SDS-PAGE and immunoblotting using specific antibodies. (C) *rrd1*Δ*/rrd2*Δ and *rrd1*Δ*/rrd2*Δ*/tpd3*Δ cells containing the plasmid PYES2[myc-TPD3] were grown to log phase in complete dropout medium. Growth of the double and triple mutant was compared by spotting equal amounts of 10-fold serially diluted cells onto synthetic complete (SC) medium plates ± 1 g/l 5-fluoroorotic acid (5-FOA). The plates were incubated for 2 d (SC) or 5 d (SC + 5-FOA) at 30 °C. Screening on 5-FOA selects for the loss of the *URA3* plasmid PYES2[myc-TPD3] and indicates dependency of viability on TPD3.

The similarity in the defect of the C subunit in the *tpd3*Δ and *rrd1*Δ*/rrd2*Δ strains raised the possibility of a physical and/or functional interaction between RRD2 and TPD3:PPH21 dimers. We therefore tested whether RRD2, TPD3, and PPH21 can form a trimeric complex in a wild-type strain, a possibility that has been suggested recently, but not proven [20]. First we analyzed myc-tagged RRD2 immunoprecipitates from lysates of the wild-type strain co-expressing HA-tagged PPH21, for associated PP2A subunits (Figure 3A) and compared it with the composition of HA-PPH21 immunoprecipitates from lysates of the same strain. The analysis revealed that approximately 5%–10% of PPH21 was associated with RRD2 in a wild-type cell. Moreover, the RRD2-bound PPH21 was unmethylated, indicating that methylation probably was not a requirement for complex formation with RRD2. This was in agreement with the association between PPH21 and RRD2 in the *ppm1*Δ strain (Figure 2A). RRD2 also associated with TPD3, but not with the regulatory subunits TAP42, CDC55, RTS1, and PPE1 (unpublished data). The lack of interaction with TAP42 was in contrast to the recent identification of TAP42:RRD1 and TAP42:RRD2 complexes and might be due to differences in experimental conditions and/or the strain background [20].

To test whether RRD2, TPD3, and PPH21 co-exist in the same complex, we immunoprecipitated myc-tagged RRD2 from lysates of a wild-type strain co-expressing HA-tagged PPH21, eluted the RRD2 complexes with the myc-epitope peptide, and re-immunoprecipitated the complexes via HA-tagged PPH21 (Figure 3B). Analysis of the HA-PPH21 immunoprecipitates with antibodies to the A subunit, TPD3, revealed that these proteins indeed are in the same multimeric complex. These findings are consistent with TPD3 and the RRD proteins playing a common role in C subunit biogenesis. To further test the functional interaction between these proteins, we deleted *TPD3* and both *RRD* genes and examined the strain viability by a proliferation assay. In line with our hypothesis, deletion of *TPD3* and both *RRD* genes was synthetically lethal (Figure 3C), providing further evidence for the essential functional and/or physical interaction of these proteins.

### Strains Impaired in C Subunit Biogenesis Display Decreased C Subunit Methylation and Increased PPE1 Binding

**Figure 4:**
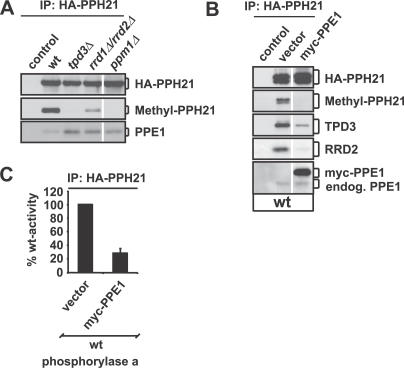
Strains Impaired in C Subunit Biogenesis Display Decreased C Subunit Methylation and Increased PPE1 Binding (A) Anti–HA-tag immunoprecipitates (IP) from lysates of wild-type (wt), *tpd3*Δ, *rrd1*Δ*/rrd2*Δ, and *ppm1*Δ cells expressing HA-tagged PPH21 were analyzed by SDS-PAGE and immunoblotting. C subunit methylation and the presence of PPE1 were analyzed using specific antibodies. (B and C) HA-PPH21 immunoprecipitates from lysates of wild-type cells expressing HA-tagged PPH21 together with myc-tagged methylesterase (myc-PPE1) (overnight induction under the control of the *GAL1* promoter), or containing an empty pYES2 vector (vector), were analyzed by SDS-PAGE/immunoblotting (B) and by phosphatase assays (C) towards phosphorylase a *(n* = 3). The blots were sequentially incubated with anti-methyl PP2A, anti-RRD2, rabbit polyclonal anti-TPD3, anti-PPE1, and anti-HA antibodies. endog., endogenous. Lanes 4 and 5 (A) and lanes 2 and 3 (B) were not adjacent on the original blot.

The 36% decreased interaction between RRD2 and the C subunit (Figure 2A) cannot explain the 75% decreased catalytic activity towards phosphorylase a in a *tpd3*Δ strain (Figure 1A), because deletion of *RRD2* reduces catalytic activity towards phosphorylase a and other P-Ser/P-Thr substrates only by about 45%–50% [13]. We therefore investigated whether the reversible methylation of the C subunit, which is required for stable holoenzyme formation [9,10], but probably also for the activation of PP2A activity [13], was also altered in the *tpd3*Δ strain (Figure 4A). Anti–HA-tag immunoprecipitates from lysates of the wild-type, *tpd3*Δ, *rrd1*Δ*/rrd2*Δ, and *ppm1*Δ strains were analyzed with the methylation-specific monoclonal antibody 2A10. No C subunit methylation was detected in the strain deleted for *PPM1* (Figure 4A, lane 5), proving the specificity of the 2A10 antibody. Surprisingly, PPH21 was almost completely unmethylated in the *tpd3*Δ strain and markedly hypomethylated in the *rrd1*Δ*/rrd2*Δ strain compared to the wild-type strain, suggesting that in the absence of TPD3 or RRD/PTPA, the catalytically impaired C subunit became either a preferred substrate for PPE1 or an unfavored substrate for PPM1.

To identify the potential cause for the decreased methylation levels in these strains, we analyzed HA-tagged PPH21 immunoprecipitates for associated PPE1 or PPM1. The yeast methyltransferase, PPM1, did not form stable complexes with the C subunit in either the wild-type or the deletion strains, suggesting that methylation occurs during a transient interaction between substrate and enzyme (unpublished data). However, deletion of either *TPD3* or *RRD1/RRD2* caused an increase in the abundance of a PPE1:PPH21 complex (Figure 4A), suggesting that the reduced methylation levels of PPH21 resulted from increased PPE1 binding (Figure 4A, lower panel). The complex formation between PPE1 and PPH21 in the *ppm1*Δ strain, however, also showed that methylation was not a prerequisite for the interaction of PPE1 with the C subunit. In summary, these data indicated that the PPE1:C subunit complex formation represents an early step in PP2A biogenesis and that interruption of the cascade causes accumulation of these complexes.

An increase in the abundance of PPE1:PPH21 complexes and demethylation of the C subunit correlated with an impaired catalytic activity in the *tpd3*Δ or *rrd1*Δ*/rrd2*Δ strains. We therefore asked whether overexpression of PPE1 in a wild-type strain would also negatively affect C subunit activity (Figure 4B and 4C). Loss of C subunit methylation by high-level overexpression of PPE1 correlated with a large increase in the amount of PPE1:PPH21 complexes, a profound reduction in the binding of PPH21 to RRD2 and TPD3 (Figure 4B), and a severely reduced PPH21 phosphatase activity towards phosphorylase a (Figure 4C). These data suggest that the impaired catalytic activity of the C subunit in a *tpd3*Δ strain was due to the increased PPE1 binding and demethylation of the C subunit, which prevented the next critical step in C subunit biogenesis.

### Deletion of PPE1 Restores C Subunit Activity in the *tpd3*Δ, but Not in the *rrd1*Δ*/rrd2*Δ Strain

**Figure 5:**
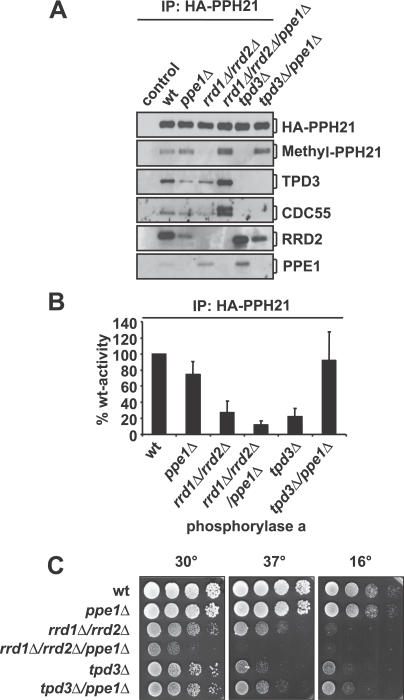
Deletion of *PPE1* Restores C Subunit Catalytic Activity in the *tpd3*Δ, but Not in the *rrd1*Δ*/rrd2*Δ Strain (A) HA-tagged PPH21 was immunoprecipitated from lysates of wild-type (wt), *ppe1*Δ, *rrd1*Δ*/rrd2*Δ, *rrd1*Δ*/rrd2*Δ/*ppe1*Δ, *tpd3*Δ, and *tpd3*Δ*/ppe1*Δ cells. The immunoprecipitates were separated by 10% SDS-PAGE and analyzed by immunoblotting with anti-HA, anti-methyl PP2A, monoclonal anti-TPD3, anti-CDC55, anti-RRD2, and anti-PPE1 antibodies. (B) Aliquots of the anti–HA-tag immunoprecipitates used in (A) were assayed for phosphatase activity using phosphorylase a as substrate (*n* = 7). (C) Logarithmically growing cultures of wild-type, *ppe1*Δ, *rrd1*Δ*/rrd2*Δ, *rrd1*Δ*/rrd2*Δ/*ppe1*Δ, *tpd3*Δ, and *tpd3*Δ/*ppe1*Δ were 10-fold serially diluted in YPD liquid medium, spotted on YPD plates, and incubated at 16 °C for 5 d, or at 30 °C or 37 °C for 3 d.

If demethylation blocks C subunit maturation, then deletion of the *PPE1* gene in a *tpd3*Δ strain might allow the biogenesis to proceed and restore the impaired catalytic activity of the C subunit. Deletion of *PPE1* in the absence of RRD1/RRD2, the essential factors for the generation of active PP2A [13], however, should, if anything, worsen the defective C subunit biogenesis. Thus, we analyzed the activity and complex composition of HA-PPH21 from a series of deletion strains (Figure 5A and 5B). As before, *tpd3*Δ and *rrd1*Δ*/rrd2*Δ strains had marked hypomethylation of PPH21. As predicted, deletion of *PPE1* not only restored C subunit methylation in these strains, but even increased it, indicating that PPE1 is responsible for the reduced amounts of methylated C subunits in these strains (Figure 5A). Importantly, *PPE1* deletion affected C subunit biogenesis in distinctly different ways in the *tpd3*Δ versus the *rrd1*Δ*/rrd2*Δ strain. In the *tpd3*Δ strain, *PPE1* deletion restored C subunit activity (*n* = 7, *p* < 0.001) towards phosphorylase a almost to the level of the wild-type strain (Figure 5B). Thus, TPD3 is not required for formation of the catalytically active C subunit.

In contrast, the RRD1/RRD2 proteins were required for the restoration of phosphatase activity: in the *rrd1*Δ*/rrd2*Δ strain, the impaired C subunit activity not only could not be rescued by *PPE1* deletion, but was further reduced (*n* = 7, *p* < 0.05) (Figure 5B). The same was true for the activity towards cdc2-phosphorylated histone H1, a substrate known to be specifically dephosphorylated by heterotrimeric complexes containing the B subunit [21] (unpublished data). The restoration of C subunit methylation in this strain, however, led to a huge increase in formation of inactive holoenzymes with TPD3 and CDC55. Of note, in these inactive complexes, a subset of CDC55 migrated with decreased electrophoretic mobility (Figure 5A). Treatment with λ-phosphatase shifted the slower form to the faster migrating form of CDC55, indicating that the altered electrophoretic mobility of CDC55 was probably due to phosphorylation (unpublished data). Interestingly, our previous analysis had revealed that in the absence of RRD1/RRD2, the level of a post-translationally modified form of CDC55 is also increased [13], raising the intriguing possibility that RRD/PTPA function regulates the modification/phosphorylation state of CDC55 and probably also of the other B-type subunit, RTS1 (unpublished data). The exacerbation of the impaired C subunit activity in the *rrd1*Δ*/rrd2*Δ strain upon deletion of *PPE1* indicated a functional and/or physical interaction between these proteins. Since we did not detect a physical interaction between PPE1 and RRD1 or RRD2 (unpublished data), the data are consistent with RRD1/RRD2 and PPE1 being in the same functional pathway without being in a complex.

The impaired C subunit activity in the *tpd3*Δ strain seems to be responsible for the severe proliferation phenotype of this strain. If the impaired activity is indeed the cause for the phenotype, then reactivation of C subunit activity upon *PPE1* deletion might alleviate the severity of the phenotype. Therefore, we measured the proliferation of these strains at different temperatures (Figure 5C). However, the *tpd3*Δ*/ppe1*Δ strain with its restored PP2A activity grew only slightly better at all tested temperatures than the *tpd3*Δ strain. We suspect that this minor improvement is due to the lack of properly targeted and regulated PP2A holoenzymes. In the *rrd1*Δ*/rrd2*Δ strain, the proliferation phenotype was aggravated upon *PPE1* deletion, despite the increase in trimeric holoenzymes, most likely due to the severe defect in catalytic activity.

### C Subunit Methylation Is Required for the Restoration of C Subunit Activity in the *tpd3*Δ*/ppe1*Δ Strain

**Figure 6:**
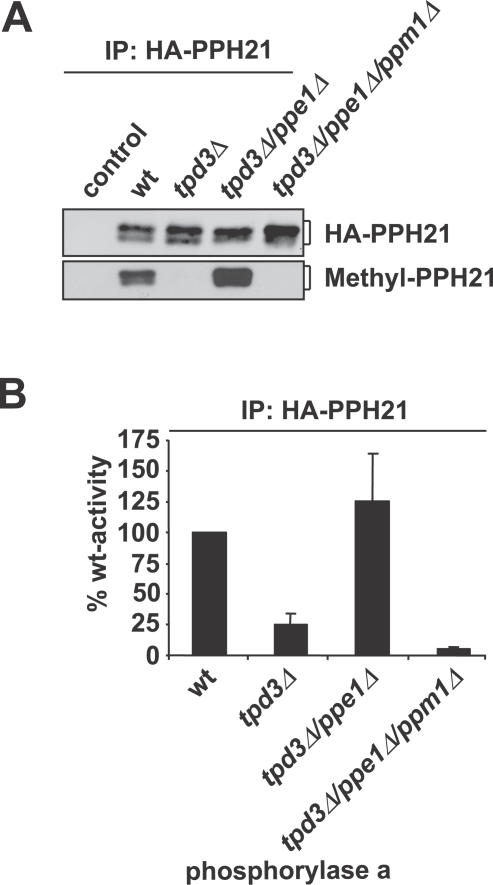
C Subunit Methylation Is Required for the Restoration of C Subunit Activity in the *tpd3*Δ*ppe1*Δ Strain HA-tagged PPH21 was immunoprecipitated from lysates of wild-type (wt), *tpd3*Δ, *tpd3*Δ*/ppe1*Δ, and *tpd3*Δ*/ppe1*Δ*/ppm1*Δ cells. The immunoprecipitates (IP) were (A) analyzed by 10% SDS-PAGE/immunoblotting and (B) assayed for phosphatase activity using phosphorylase a as substrate (*n* = 7).

The increase of C subunit methylation in the *tpd3*Δ*/ppe1*Δ strain correlated with a restoration of the impaired catalytic activity, indicating a potential requirement for methylation in the C subunit activation process, a role to which our previous data had already pointed [13]. To test this hypothesis, we deleted the *PPM1* gene in the *tpd3*Δ*/ppe1*Δ strain and analyzed the methylation state and catalytic activity towards phosphorylase a in this strain (Figure 6A and 6B). Loss of C subunit methylation in this strain caused a huge decrease in catalytic activity (*n* = 7, *p* < 0.001) (Figure 6B), underscoring the importance of methylation for C subunit activation. Thus, PPE1 probably delays progression of the biogenesis cascade in the *tpd3*Δ strain by keeping the C subunit in an unmethylated state.

## Discussion

**Figure 7:**
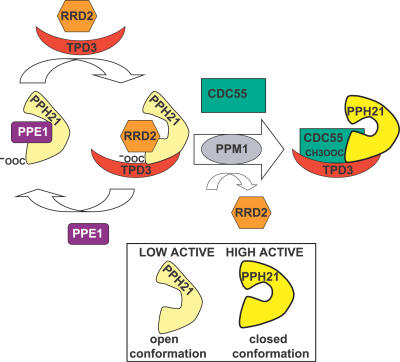
Model: RRD/PTPA-Dependent Generation of Active PP2A C Subunit Is Coupled to Holoenzyme Assembly and Regulated by Methyl-Esterase/Transferase Enzymes

Here we identify a critically important functional and physical interaction between RRD2 and the structural subunit TPD3 in C subunit maturation. RRD2/TPD3 interacts preferentially with the demethylated and inactive form of the C subunit by targeting the PPH21:PPE1 complex. PPE1, therefore, may fulfill a surveillance function of TPD3/RRD2-dependent C subunit maturation by preventing untimely C subunit methylation and hence the premature generation of active C subunit and holoenzyme assembly (see model, Figure 7).

The catalytic subunit of a multisubunit enzyme like PP2A achieves substrate specificity by complex formation with regulatory subunits [22]. The combinatorial assembly of holoenzymes from a large pool of distinct regulatory subunits generates a great number of different substrate-specific complexes and explains how a single catalytic subunit is able to perform so many functions. Inherent to the multisubunit enzyme architecture, however, is the problem of how the promiscuous catalytic activity of the free catalytic subunit is kept in check until holoenzymes form. Recently, we obtained experimental evidence for a potential control mechanism, which involves synthesis of the PP2A catalytic subunit in a low-activity form that requires the functional interaction with RRD/PTPA for the switch into an active and specific enzyme [13]. Here we show that the scaffolding subunit TPD3 functionally and physically interacts with RRD2 in the generation of the active and specific PP2A C subunit, indicating that C subunit maturation is coupled and coordinated with holoenzyme assembly.

Biochemical analyses indicated that in vitro, binding of the A subunit to the C subunit decreases the phosphatase catalytic rate and increases substrate specificity [7]. However, the effects of PP2A A subunit binding on C subunit catalytic properties were determined with C subunits isolated from catalytically active PP2A holoenzymes, because the free C subunit does not seem to exist in substantial amounts in vivo. On the basis of these in vitro observations, it has been assumed that loss of the A subunit in vivo will leave behind the monomeric C subunit with high and promiscuous catalytic activity. The data presented here demonstrate that this assumption is incorrect. Our analysis of the *tpd3*Δ strain indicates that the A subunit provides an additional function besides narrowing the substrate specificity of an otherwise promiscuous C subunit. As evidenced by genetic and physical interactions, the A subunit also cooperates with RRD/PTPA in the generation of the active C subunit in vivo.

What is the regulatory role of TPD3 in this process? TPD3 formed a multimeric complex with RRD2 and PP2A C subunits. This complex appears to be essential for PP2A biogenesis and differs in several aspects from B-type subunit-containing holoenzymes. In contrast to B-type subunits, RRD2 interacted preferentially with the unmethylated C subunit, suggesting methylation state-dependent regulation of RRD2:C subunit interaction. We found, consistent with this hypothesis, that deletion of *PPE1* (as well as PPM1 overexpression; unpublished data) decreased the levels of RRD2:TPD3:C subunit complexes. RRD2:TPD3:PPH21 holoenzyme assembly constitutes the first example of a PP2A complex whose stability is regulated in an opposite manner to other PP2A holoenzymes, namely by demethylation rather than methylation. The binding preference of RRD2 for the demethylated C subunit suggests an important role for PPE1, the enzyme catalyzing the demethylation, as well as for the carboxyl terminus itself in the interaction with RRD2. In accordance with our prediction, a C subunit mutant lacking the carboxy-terminal nine amino acids including the site of methylation did not bind RRD2, and the monoclonal antibody 7A6 directed to the C subunit carboxyl terminus did not co-precipitate RRD2 (unpublished data), indicating that the C subunit carboxyl terminus seems to be essential for the interaction (directly or indirectly) with RRD2. The impaired catalytic activity of the deletion mutant towards phosphorylase a (unpublished data) provided further evidence for the functional cooperation between the C subunit carboxyl terminus and RRD2.

So far, no obvious phenotype has been identified following deletion of *PPE1* in yeast [10], raising questions about PPE1′s role and importance in vivo as the opposing enzyme of the PP2A methyltransferase, PPM1. Whereas PPM1 function (C subunit methylation) is a prerequisite for stable complex formation with B-type subunits [9,10], the PPE1 function in the regulation of trimeric PP2A holoenzymes remained obscure because conflicting data exist on its ability to demethylate the C subunit once it is bound to B-type subunits [23,24]. Thus, it is currently thought that PPE1 regulates PP2A holoenzyme assembly with B-type subunits by shifting the equilibrium between the demethylated and the methylated AC core dimer. Our data indicate a role for PPE1 earlier in the biogenesis of PP2A, namely in the regulation of complex assembly with RRD2 rather than with B-type subunits. PPE1 appears to be required for stable complex formation between the C subunit and TPD3:RRD2, and counteracts the premature methylation of the C subunit that has not yet been activated by TPD3:RRD/PTPA. Defects in C subunit biogenesis, for example by deletion of either the *TPD3* or *RRD* genes, caused the accumulation of complexes of PPE1 and the C subunit in its low-activity conformation. Thus, PPE1 seems to constitute a surveillance mechanism that prevents the premature generation of the active C subunit in the absence of the scaffolding subunit TPD3, the major constituent of PP2A holoenzymes, or premature holoenzyme assembly in the absence of the essential activator RRD/PTPA. PPE1 seems to achieve this function in two ways, by competing with RRD2 for the binding to the C subunit and, more importantly, by keeping the C subunit in its demethylated state and thereby preventing untimely methylation that would otherwise complete the C subunit activation and promote complex formation with B-type subunits. In line with this prediction, deletion of *PPE1* in a *tpd3*Δ strain overcame the PPE1-dependent block of C subunit maturation despite the inability for holoenzyme formation, and deletion of *PPE1* in the *rrd1*Δ*/rrd2*Δ strain increased dramatically the levels of trimeric holoenzymes with CDC55 despite the fact that the C subunit was in its inactive conformation.

How do defects in C subunit biogenesis lead to the increased interaction between PPE1 and the C subunit? At the moment, our data do not allow us to distinguish between several potential scenarios of PPE1 function. In the first scenario, PPE1 constitutes one of the first steps of C subunit biogenesis, interacting with the newly synthesized C subunit in its low-activity conformation. Interruptions of the biogenesis cascade would then cause the accumulation of complexes from earlier steps because dissociation of these complexes is interlocked with formation of the subsequent complex, e.g., the TPD3:RRD2 complex. In the second scenario, PPE1 plays a more active role by interacting with and demethylating C subunits that have not yet been activated by RRD/PTPA. PPE1 could achieve this by a substrate preference for any C subunit in its low-activity conformation, including those that are newly synthesized and inactive. Our results from PPE1 overexpression experiments (but also the finding of increased PPE1 binding levels in the *tpd3*Δ and *rrd1*Δ*/rrd2*Δ strains) are also consistent with a third mechanism in which PPE1 targets the catalytically active C subunit, demethylates it, and through its binding to catalytic site residues, converts the C subunit into the low-activity conformation, which subsequently requires RRD/PTPA as well as C subunit methylation for recycling in the biogenesis cascade.

This biogenesis mechanism appears to function in mammals as well as yeast. Consistent with an affinity of PPE1 for the inactive C subunit, we were able to identify and isolate the mammalian PPE1 ortholog, PME1, by its increased interaction with catalytically defective C subunit mutants [25], and PME-1 was recently co-purified with inactive PP2A [24]. Interestingly, several of the human C subunit active-site mutant proteins also bind increased levels of PTPA (the mammalian ortholog of RRD2) as well as α4/TAP42 [13], suggesting a binding preference of both PTPA and α4/TAP42 for the inactive conformation of the C subunit. The increased binding of these molecules to C subunit mutants that are unable to fold into the catalytically active conformation and complete the biogenesis suggests that they might play key roles in the early steps of C subunit biogenesis.

TAP42 is an essential protein [17] known to interact with the catalytic subunits of the PP2A family of phosphatases, including PPH21, PPH22, SIT4, and PPH3 [26]. A recent study identified RRD1 and RRD2 as part of the TAP42:phosphatase complexes [20] in the W303 strain, and confirmed earlier findings that RRD/PTPA proteins are required for phosphatase activity [13,27]. In addition, these authors detected a complex between the RRD proteins and TPD3, but the significance of this complex remained unclear. Our results now identify the functional and physical interaction between RRD2 and TPD3 in C subunit biogenesis. In contrast to their findings, we were unable to detect significant complex formation between RRD2 and TAP42 in the BY strain. Thus, the observed differences may be due to the different genetic backgrounds of the W303 and BY strains. It will be interesting to test whether and how the differences in TAP42:RRD complexes affect PP2A biogenesis in these strains.

By what mechanism might RRD/PTPA regulate C subunit biogenesis? We showed recently that loss of RRD proteins results in the generation of PP2A and SIT4 catalytic subunits with impaired catalytic activity, indicating alteration of the active site. Thus, we hypothesized that RRD/PTPA might play a role in the generation of the native conformation of the PP2A and SIT4 active sites. In line with our assumption, preliminary evidence has been provided for a potential peptidyl-prolyl *cis/trans* isomerase (PPIase) activity of RRD/PTPA [28] that is stimulated by ATP/Mg^2+^ and, which could be responsible for switching the C subunit into the active conformation. Evidence for a potential PPIase activity was further provided by the recent structure determination of RRD/PTPA in complex with a proline-containing PPIase peptide substrate [29]. No ATP binding site, however, could be identified in the RRD/PTPA structure [29,30]. Interestingly, SSB2, a ribosome-associated chaperone that possesses ATPase activity and plays a role in the folding of newly synthesized proteins has been found just recently in a complex with TPD3 [31]. It is tempting to speculate that the energy necessary for the PTPA-catalyzed conformational change in the C subunit might be provided by ATP hydrolysis through the TPD3-bound SSB2. It would also provide a potential explanation for our finding of the functional and physical interaction between RRD/PTPA and TPD3. Chao et al. [32], however, identified an ATP binding pocket in their structure of PTPA and showed that PTPA and the PP2A A-C dimer together constitute a composite ATPase, whose activity is required for the transient change of PP2A into a phosphotyrosine (P-Tyr)-specific phosphatase, at least in vitro. How the loss of this transient P-Tyr–specific activity would lead to the huge decrease of the P-Ser/P-Thr–specific activity of trimeric PP2A holoenzymes that is observed in the *rrd1*Δ*/rrd2*Δ strain [13], or now in the *rrd1*Δ*/rrd2*Δ/*ppe1*Δ strain, is unclear. The requirement for an activator protein, however, is not unique to the PP2A family of phosphatases. Inhibitor-2 (I-2), a highly conserved protein, interacts with the catalytic subunit of protein phosphatase 1 (PP1) and inhibits its catalytic activity in vitro [33]. Deletion of *GLC8,* encoding the yeast I-2 ortholog, however, leads to a reduction and not to an increase of GLC7 catalytic activity, as would have been expected for the loss of an inhibitor [34]. The catalytically inactive PP1 in complex with I-2 is activated upon phosphorylation of I-2, which probably induces a conformational change in PP1, suggesting a function of inhibitor-2 as a PP1 chaperone [35,36]. Whether the I-2 chaperone function, and thus the generation of active PP1, is also coupled to and coordinated with PP1 holoenzyme assembly is currently unknown, but—given our present results—this is a likely possibility.

Deletion of RRD/PTPA affected the post-translational modification state of the yeast B-type subunits CDC55 [13] and RTS1 (unpublished data), suggesting the intriguing possibility that phosphorylated B-type subunits are substrates of RRD/PTPA complexes in vivo. RRD/PTPA may represent a unique species of PP2A regulatory subunits, with a very restricted number of (potentially even P-Tyr) phosphorylated substrates. In this way, the generation of the active C subunit would be tightly coupled to the formation of substrate-specific holoenzymes. Cells could avoid potential damage from the highly active but untargeted C subunit by coupling the RRD/PTPA-dependent maturation process to the interaction of the C subunit with the scaffolding subunit TPD3, which in turn constitutes a prerequisite for holoenzyme assembly with B-type subunits (model, Figure 7). The order of events seems to be under the surveillance of the PP2A methylesterase that preferentially binds to the C subunit in its low-activity conformation, consistent with PPE1′s increased affinity for inactive C subunit mutants. Demethylation of the C subunit by PPE1 is required for the efficient interaction with RRD2, and at the same time, demethylation prevents interaction with B-type subunits. TPD3 and RRD2 are both required for the release of PPE1 and the generation of the active and specific C subunit. The RRD2-dependent activation of the phosphatase catalytic activity is coupled to the assembly with B-type subunits by targeting the RRD2:TPD3:C subunit complexes to their potential substrates, phosphorylated B-type subunits. The process is completed by C subunit methylation destabilizing the interaction with RRD2 and, at the same time, stabilizing the one with B-type subunits. This model provides an explanation for how cells deal with the inherent danger of the multisubunit enzyme architecture, namely the untargeted and unrestricted catalytic activity of the free C subunit.

## Materials and Methods

### Yeast strains, gene disruption, and growth media.

Saccharomyces cerevisiae strains used in this study are listed in Table 1. The double-deletion strains *cdc55*Δ*/rts1*Δ and *tpd3*Δ*/ppe1*Δ were generated by PCR-based gene targeting [37] from the single-deletion strains *rts1*Δ and *tpd3*Δ, respectively. The *rrd*Δ*/rrd2*Δ*/ppe1*Δ strain was obtained by mating the strains *rrd1*Δ*/rrd2*Δ *(MAT**a**)* and *ppe1*Δ (*MATα,* EUROpean *Saccharomyces Cerevisiae* ARchive for Functional analysis [Euroscarf], http://web.uni-frankfurt.de/fb15/mikro/euroscarf/index.html), followed by sporulation and tetrad dissection. The *tpd3*Δ*/ppe1/*Δ*ppm1*Δ strain was obtained in the same way, by mating the strains *tpd3*Δ*/ppe1*Δ *(MAT**a**)* and *ppm1*Δ (*MAT*α; Euroscarf). After phenotypic analysis, gene deletions were confirmed by PCR and by immunoblotting analysis of yeast lysates with specific antibodies.

**Table 1:**
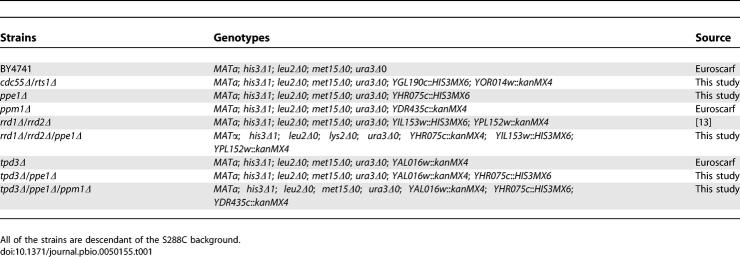
Yeast Strains

The strains were grown at 30 °C in rich medium (YPD) or complete minimal (CM) dropout medium supplemented with 2% glucose or 2% galactose and 0.1% raffinose.

### Plasmids.

Plasmids used for this study are summarized in Table 2. Detailed cloning strategies and information of the individual constructs can be obtained upon request.

**Table 2:**
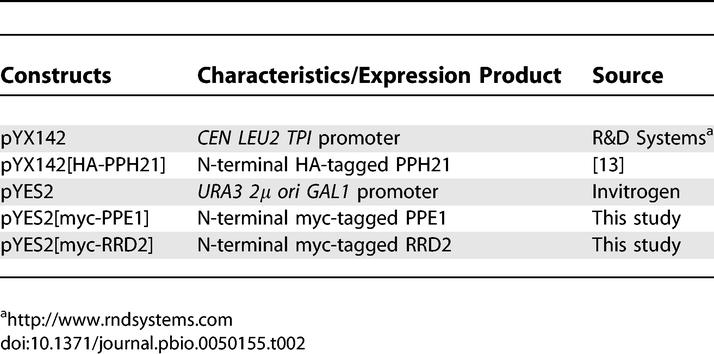
Yeast Constructs

### Synthetic lethality analysis.

Analysis of 74 tetrads obtained by mating *rrd1*Δ*/rrd2*Δ (*MAT**a*** [13]) with the *tpd3*Δ (*MATα*; Euroscarf) strain revealed the absence of any viable *rrd1*Δ*/rrd2*Δ*/tpd3*Δ triple-deletion mutant. To corroborate these data, the heterozygous diploid strain was transformed with the plasmid pYES2[myc-TPD3] (2 μm, *URA3*) and sporulated. The spores were dissected, analyzed phenotypically for the presence of resistance and auxotrophic markers (Kan^R^, His^+^, and Ura^+^), and tested for protein expression by immunoblotting. The Ura^+^ *rrd1*Δ*/rrd2*Δ*/tpd3*Δ mutants were spotted on synthetic complete (SC) medium plates ± 1 g/l 5-fluoroorotic acid (5-FOA).

### Yeast cell extracts, immunoprecipitation, and immunoblotting.

The preparation of whole-cell yeast extracts was carried out as previously described [13]. HA- or myc-tagged proteins were immunoprecipitated by using anti-HA (12CA5) or anti-myc (4A6; Upstate, http://www.upstate.com) antibodies cross-linked to BSA-coated protein A sepharose beads (Pharmacia Biotech [GE Healthcare], http://www.gehealthcare.com/usen/index.html) as described previously [38]. To avoid stripping of the immunoblots after antibody incubation, immunoprecipitates were split into equivalent aliquots after boiling in sample buffer and analyzed by 10% SDS-PAGE. Detection of the co-immunoprecipitated proteins was performed using the monoclonal antibodies anti-HA (clone 16B12; Covance Research Products, http://www.crpinc.com), anti-methyl-PP2A (clone 2A10; Upstate,http://www.upstate.com), anti-RRD2 [13], and anti-TPD3 (raised against residues 65–597, clone 5G2), and the rabbit polyclonal antibodies anti-CDC55 [9], anti-PPE1 (raised against full-length PPE1), anti-PPH21 [13], anti-TAP42 (raised against full-length TAP42), and anti-TPD3 [9].

For the experiment in Figure 3B, peptide competition and re-immunoprecipitation was carried out as follows: 200 optical density at 600 nm (OD_600_) logarithmically growing yeast cells were used for myc-RRD2 immunoprecipitation. Immunoprecipitates were washed and eluted by two sequential incubations for 5 min at 30 °C in the presence of 100 or 50 μg/ml (first and second elution, respectively) of the myc peptide (MEQKLISEEDL) in TBS buffer. The eluates were pooled and used for the second immunoprecipitation, carried out in the same way as before, except using anti-HA antibody cross-linked to protein A sepharose beads.

### Disruption of PPH21 complexes and isolation of the monomeric catalytic subunit.

Monomeric HA-PPH21 was isolated by immunoprecipitation of yeast lysates, which had been treated with a basic pH shift to disrupt protein complexes, followed by neutralization. The lysates were generated as described before by [13] except that the yeast lysis buffer contained 10 mM instead of 50 mM Tris-Cl. The basic pH shift (pH 11.5) was obtained by adding 6.25 μl of triethylamine to 500 μl of yeast cell lysate. After 5 min incubation at room temperature, the reaction was neutralized by adding 330 μl of 0.1 N HCl and was used for immunoprecipitation experiments/phosphatase assays as described in the next paragraph.

### Protein phosphatase assays.

Phosphatase activity of PPH21 and PPH22 immunoprecipitates was assayed towards ^32^P-labeled phosphorylase a, ^32^P-labeled histone H1, and para-nitrophenyl phosphate (pNPP; Sigma, http://www.sigmaaldrich.com). Details about ^32^P-labeled phosphorylase a and ^32^P-labeled histone H1 preparation were described previously (see [38]).

To determine the phosphatase catalytic activity of HA-PPH21 or HA-PPH22 towards phosphorylase a, histone HI, or pNPP, the equivalent of 50 OD_600_ logarithmically growing cells were used for native whole-cell lysate preparation as previously described [13]. The lysates were used for immunoprecipitation (1 h at 4 °C, under rocking) with 40 μl of anti-HA (12CA5) antibody cross-linked to BSA-coated protein A sepharose beads (1:1 suspension in PBS). For PPH21 endogenous activity (Figure S3), the lysate was incubated with 2 μl of rabbit polyclonal anti-PPH21 serum (or preimmune serum for control immunoprecipitation) for 30 min followed by another 30 min in the presence of 40 μl of BSA-coated protein A sepharose beads (1:1 suspension in PBS) at 4 °C. The immunoprecipitates were washed one time with yeast lysis buffer and three times with TBS. To test the catalytic activity of PPH21/PPH22 complexes (or the monomeric C subunit) towards ^32^P-phosphorylase a or pNPP, one fourth of the immunoprecipitate (resuspended in 400 μl of TBS) was used for activity determination. For ^32^P-histone HI activity analysis, 1/32 of the immunoprecipitate was used. In addition, one fourth of each of the immunoprecipitates was used to normalize the PPH21 amounts (see below). Further steps were performed as previously reported in detail for PP2A isolated from mammalian cells [38].

The unspecific activity (background) was determined by using a wild-type strain containing an empty vector (or for endogenous PPH21, a wild-type strain incubated with PPH21 preimmune serum) and was subtracted from each point determination.

The assay values (average of at least three independent experiments) are presented as a percentage of the wild-type strain activity, which was set as 100%. For this, the values were normalized to the amount of immunoprecipitated PPH21/PPH22 as determined by immunoblot and densitometer analysis using the Personal Densitometer SI (Molecular Dynamics [GE Healthcare], http://www.gehealthcare.com/usen/index.html) or the Odyssey Infrared Imaging System (LI-COR, http://www.licor.com). We included for the assay calculations only quantifications that revealed levels of HA-PPH21/PPH22 that did not differ by more than 5.5-fold from the wild-type reference (only the immunoblots for Figure 4C were analyzed with conventional HRP detection). Moreover, we repeated the quantification if C subunit levels differed more than 5.5-fold, as follows: the remaining aliquots of the immunoprecipitates were reanalyzed after adjustment based on the rough quantification of the first immunoblot and analyzed by SDS-PAGE/immunoblot analysis as described above.

### Statistical analysis.

Data are presented as mean values ± standard deviation (SD), and were analyzed using the Student *t*-test. Differences with *p*-values less than 0.05 were considered statistically significant.

## Supporting Information

Figure S1 Loss of TPD3 Leads to a Decreased Serine/Threonine Phosphatase Activity and Altered Substrate Specificity of the PP2A C Subunit PPH22 (A) The catalytic activity of anti-HA immunoprecipitates from lysates of wild-type (wt), *tpd3*Δ, *rrd1*Δ*/rrd2*Δ, and *cdc55*Δ*/rts1*Δ cells expressing HA-tagged PPH22 was analyzed by phosphatase assays towards phosphorylase a (*n* = 4) or (B) para-nitrophenyl phosphate, pNPP (*n* = 4).
(85 KB PDF)

Figure S2 The PP2A Serine/Threonine Phosphatase Activity in the Absence of TPD3 or RRD1/2 Is Decreased Independently of the Substrate Concentration The catalytic activity of HA-PPH21 immunoprecipitates from lysates of wild-type (wt), *tpd3*Δ, *rrd1*Δ*/rrd2*Δ, and *cdc55*Δ*/rts1*Δ cells expressing HA-tagged PPH21 was analyzed by phosphatase assays towards phosphorylase a (*n* = 4). The catalytic activity was determined for each strain by using three different substrate concentrations of phosphorylase a (1, 10, and 100 μM).
(105 KB PDF)

Figure S3 The Serine/Threonine Phosphatase Activity of Endogenous PPH21 Is Decreased in the Absence of TPD3 (A) Endogenous PPH21 was immunoprecipitated from wild-type (wt) and *tpd3*Δ strains with a PPH21 polyclonal antibody, and analyzed by SDS-PAGE and immunoblotting against PPH21 and RRD2. Preimmune serum was used for the control immunoprecipitation.
(B) An aliquot of the immunoprecipitates was tested for phosphatase activity towards phosphorylase a (*n* = 4). The asterisk indicates the heavy chain of the anti-PPH21 antibody and other antibodies present in the rabbit serum.
(163 KB PDF)

Figure S4 A Basic pH Shift Promotes Dissociation of PP2A Complexes Untreated cell lysates (untreated/control) and cell lysates after a basic pH shift/neutralization step (treated) from a HA-PPH21–expressing wild-type (wt) (upper two panels) or *tpd3*Δ strain (lower two panels) were analyzed by 5%–10% sucrose gradient sedimentation (Beckman XL-90, rotor Sw60Ti, 4 h at 54,000 rpm, 4 °C). Twenty-five consecutive fractions were collected (from the top to the bottom) and analyzed by SDS-PAGE and immunoblotting. The sucrose gradient fractions containing the sedimentation markers carbonic anhydrase (29 kDa), albumin (66 kDa), aldolase (158 kDa), and catalase (232 kDa) are indicated on the top with arrows. The membranes were incubated with an anti-HA antibody.
(2.9 MB PDF)

Figure S5 The Serine/Threonine Phosphatase Activity of the Monomeric C Subunit Isolated from a *tpd3*Δ or *rrd1*Δ*/rrd2*Δ Strain Is Decreased Independently of the Substrate Concentration Monomeric HA-PPH21 was immunoprecipitated from lysates of wild-type (wt), *tpd3*Δ, *rrd1*Δ*/rrd2*Δ, and *cdc55*Δ*/rts1*Δ cells that were treated with a basic pH shift to disrupt protein complexes followed by a neutralization step (see Materials and Methods for details). The serine/threonine phosphatase activity of the HA-tag immunoprecipitates was tested towards three different concentrations of phosphorylase a (1, 10, and 100 μM) (*n* = 3).
(108 KB PDF)

## Abbreviations

HA: hemagglutinin
I-2: inhibitor-2
pNPP: para-nitrophenyl phosphate
PP1: protein phosphatase 1
PP2A: protein phosphatase 2A
PPE1: protein phosphatase 2A methylesterase 1
PPM1: protein phosphatase methyltransferase 1
P-Ser/P-Thr: phospho-serine/phospho-threonine
PSTK: protein serine/threonine kinase
PSTP: protein serine/threonine phosphatase
PTPA: protein phosphatase 2A phosphatase activator
RRD: rapamycin-resistant deletion
