# Supplementary material for: Generation of Active Protein Phosphatase 2A Is Coupled to Holoenzyme Assembly
Source: PLoS Biol. 2007 Jun 5;5(6):e155. doi: 10.1371/journal.pbio.0050155 (PMC1885835; doi:10.1371/journal.pbio.0050155)

Suppl. Figure S5

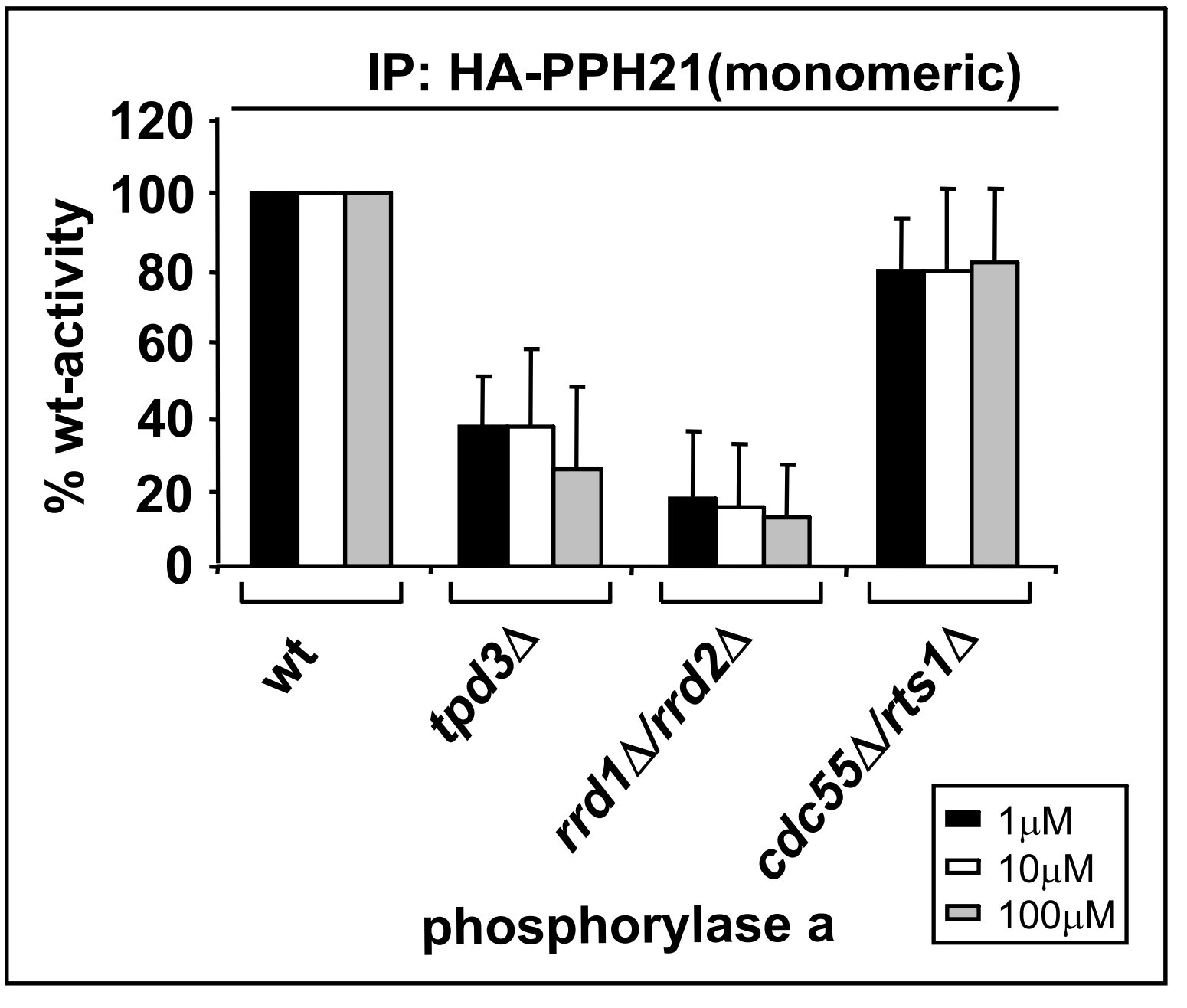

Supplement: Supplementary file 5 [file pbio.0050155.sg005.pdf]
